# Filamentous Biopolymers on Surfaces: Atomic Force Microscopy Images Compared with Brownian Dynamics Simulation of Filament Deposition

**DOI:** 10.1371/journal.pone.0007756

**Published:** 2009-11-04

**Authors:** Norbert Mücke, Konstantin Klenin, Robert Kirmse, Malte Bussiek, Harald Herrmann, Mathias Hafner, Jörg Langowski

**Affiliations:** 1 Division Biophysics of Macromolecules, German Cancer Research Center, Heidelberg, Germany; 2 Department of Molecular Genetics, German Cancer Research Center, Heidelberg, Germany; 3 Institute of Molecular and Cell Biology, University of Applied Sciences Mannheim, Mannheim, Germany; Griffith University, Australia

## Abstract

Nanomechanical properties of filamentous biopolymers, such as the persistence length, may be determined from two-dimensional images of molecules immobilized on surfaces. For a single filament in solution, two principal adsorption scenarios are possible. Both scenarios depend primarly on the interaction strength between the filament and the support: i) For interactions in the range of the thermal energy, the filament can freely equilibrate on the surface during adsorption; ii) For interactions much stronger than the thermal energy, the filament will be captured by the surface without having equilibrated. Such a ‘trapping’ mechanism leads to more condensed filament images and hence to a smaller value for the apparent persistence length. To understand the capture mechanism in more detail we have performed Brownian dynamics simulations of relatively short filaments by taking the two extreme scenarios into account. We then compared these ‘ideal’ adsorption scenarios with observed images of immobilized vimentin intermediate filaments on different surfaces. We found a good agreement between the contours of the deposited vimentin filaments on mica (‘ideal’ trapping) and on glass (‘ideal’ equilibrated) with our simulations. Based on these data, we have developed a strategy to reliably extract the persistence length of short worm-like chain fragments or network forming filaments with unknown polymer-surface interactions.

## Introduction

The flexibility of filamentous biopolymers reflects important aspects of their biological function. Probably the most extensively studied example is the bending capability of DNA for its importance in the packaging of the genome into nucleosomes, the chromatin fiber and chromosomes [1], [2]. Studies of the properties for many other filamentous biopolymers have been somewhat in the shadow of the DNA work, but they are using principally very similar concepts. The elasticity of cytoskeleton constituents such as actin filaments, microtubules, and intermediate filaments (IFs) determines the shape and mechanical properties of the cell. While the implication of the nanomechanical properties of IF proteins for their biological functions has been realized in many cases (for review see e.g. [3], these properties are only beginning to be understood.

One fundamental parameter that characterizes the conformation of a long polymer is its bending rigidity, which will determine how stiff the polymer behaves against external forces. The bending rigidity is generally expressed as the persistence length *L_p_*. This is the length after which the chain “loses memory” of its initial direction due to thermal fluctuations. For instance, actin filaments have a persistence length of approx. 10 to 20 µm, for microtubules it is 300-fold higher [4]. The persistence length of vimentin IFs is about 20 times lower [5]. To measure the persistence length of vimentin IFs, we employed atomic force microscopy (AFM). In this procedure [5], it was necessary to find conditions for the binding of vimentin filaments to the support that allowed the filament to equilibrate on the surface. The distinction between conditions of surface equilibration (SE) and surface trapping (ST) presented an important challenge for the interpretation of AFM data.

Here we compare quantitative evaluations of AFM images taken under different conditions of filament-surface interaction with computer simulations of the polymer deposition. We demonstrate that it is possible to distinguish between SE and ST conditions, even if the bending flexibility of the filament is not known a priori.

### Theoretical considerations

The persistence length *L_P_* of a polymer chain is defined as the correlation length of the direction of the chain measured along its contour in three dimensions [6], [7]

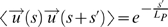
(1)


Here 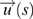
is the unit tangent vector of the chain; *s* and *s+s′* the positions along the chain contour. The angular brackets indicate the thermal average over all positions and chain conformations. Molecules shorter than *L_p_* behave approximately like a rigid rod, while longer chains show significant internal flexibility.

The bending elasticity *A* - the energy required to bend a polymer segment of unit length over an angle of one radian - is related to the persistence length by *L_P_ = A/k_B_T*, *k_B_* being the Boltzmann's constant and *T* the absolute temperature. The energy required to bend two segments of the chain of length *l* by an angle *Θ* with respect to one another is [6]

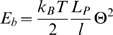
(2)


The persistence length of a polymer chain can be determined from eq. (1), averaging over a large number of chain contours. Many studies have used two-dimensional images of polymer chains on surfaces for determining their bending elasticity or permanent bending, e.g. for DNA molecules imaged by electron (EM) [8] or atomic force microscopy (AFM) [9]–[12], or for intermediate filaments imaged by AFM [5], [13], [14]. In all of these cases some assumptions must be made as regards the relation between the three-dimensional structure of a polymer chain and its two-dimensional image, which strongly depends on the polymer-surface interaction. Two extreme cases have generally been considered: i) two-dimensional equilibration, where the polymer chain interacts rather weakly with the surface and can relax to a two-dimensional equilibrium conformation, and ii) so-called ‘trapping’ where the polymer binds irreversibly to the surface, starting at a few contact points from where complete adsorption is propagated.

In the first case, when the polymer conformation is equilibrated in two dimensions, the adsorbed polymer can be treated with exactly the same formalism as the one moving free in solution, except that the chain is confined to one plane. In the case of trapping, i.e. strong polymer-surface interaction, it is generally assumed that the chain conformation on the surface is a projection of its three-dimensional conformation to the plane. This model proved particularly useful for long polymer chains [5], [9], [10], [15]. However, this approximation is too simplistic to understand the chain deposition process in detail, since significant conformational changes can occur after the chain has made contact with the surface. Thus, numerical simulations need to be done and compared with experiments performed with polymer chains of various lengths. This may allow us to connect the two-dimensional (2D) conformation of a polymer chain trapped on a surface reliably with its three-dimensional (3D) structure free in solution. Since the interaction with the surface is assumed to be very strong, its details do not influence the final conformation of the chain on the surface. This offers the advantage that fundamental physical properties of the polymer such as its persistence length can be obtained without regarding the details of surface interaction, in contrast to a scenario where some surface mobility is encountered.

Strong biopolymer surface interactions have already been observed and analyzed [5], [9], [10], [15]. For long three-dimensional chains and strong surface interactions a 3D-to-2D projection model was assumed and a broad distribution of the calculated mean-square end-to-end-distances for long DNA-fragments was reported [9], [10]. In earlier work [5], we used the same model but for shorter fragments with a broad distribution of contour lengths. The persistence length calculated in that work for longer segments of about *3L_P_* gave results comparable with filaments which were allowed to equilibrate on the surface. In contrast, for short segments the calculations failed. Consequently, a more detailed analysis was indispensable. Here we now develop a model that describes ideal trapping conditions for the whole chain.

We performed Brownian dynamics simulations of flexible chain deposition onto a flat surface and compared the resulting chain conformations with experimental data gathered on vimentin filaments with AFM. The procedure that we report here enables us to predict the 3D conformational properties even of short chains (at or below the persistence length) from the 2D images.

### Brownian Dynamics simulations

Brownian Dynamics (BD) simulations were done with *corchy++*, a BD and Monte-Carlo simulation package for linear and circular worm-like chains (WLC) described in earlier publications [16], [17]. The polymer was approximated by a chain of beads connected through straight elastic segments, each of which carried a local reference frame. The energy of the system was given by harmonic potentials with respect to: (i) the angles between consecutive segments, (ii) the twist angles between consecutive reference frames, and (iii) the deviations from the equilibrium segment length. Excluded-volume or other long range intra-chain interactions were neglected, which is justified for these chains lengths according to the theory developed by Yamakawa and Stockmayer [[18]]. Each step in the BD trajectory was performed according to the algorithm of Ermak and McCammon [19] with second order corrections [20]. The hydrodynamic interactions between the beads were given by the Rotne-Prager tensor [21]. The initial polymer conformation was obtained by a Monte Carlo procedure, in which the polymer was represented as a linear chain of straight segments with a fixed length. The energy parameters were the same as in the BD model. A trial Monte Carlo step was performed by, subdividing the chain into two parts at a random joint, and one of the parts was rotated about a random axis that passed through this joint. The angle of rotation was uniformly distributed in an interval (-Θ,Θ), where Θ was chosen in such a way that the acceptance rate was approximately 50%. The steps were accepted or rejected according to the classical Metropolis criterion. Monte Carlo steps were done successively until the chains reached the mean-square end-to-end distance expected for a relaxed chain.

This initialized conformation was relaxed in the BD force field to match the equilibrium distribution over the segment lengths. Trapping on a surface (ST) was modeled in the following way. The position of the surface was assumed to be at the horizontal plane *z = 0*. At *t = 0*, the chain was positioned in space so that its lowest joint lay at the trapping surface. Whenever during the simulation any other joint passed through the surface, its z-coordinate was set to 0 and its position became fixed for the rest of the simulation run. The simulation continued until all the joints were fixed. The hydrodynamic interactions between the chain and the surface were neglected. In the case of ‘weak’ interaction (SE), the same procedure was applied with the exception that only the z-coordinate of the trapped joints became fixed, while 2D movement was still allowed.

For historical reasons, we used parameters corresponding to double helical B-DNA. Since the length scale is taken relative to the persistence length, this can easily be rescaled for other WLC. Thus, the persistence length for B-DNA was *L_P_* = 50 nm, the equilibrium segment length *l_0_* = 1.67 nm (30 segments per one persistence length), and the total length of the chain *L* = 200 nm = *4L_P_*. For modeling the hydrodynamic radius, each 1.67 nm segment was covered with a bead of radius *r_b_* = 1.33 nm (so that the neighboring beads were overlapping). The simulation time step was *Δt* = 20 ps, the torsional rigidity *C* = 2.5⋅10^−19^ erg cm, the temperature *T* = 298 K. The elastic stretching modulus was arbitrarily chosen to be 250 pN, in order to provide reasonable computational time.

## Materials and Methods

### Preparation of intermediate filaments

The cloning and expression of recombinant human vimentin was done as described [22]–[24]. The stepwise dialysis of proteins into low salt buffers and subsequent assembly experiments were done as described [5].

### Atomic force microscopy

Assembled filaments were deposited onto glass modified to be hydrophilic or freshly cleaved mica in 2 mM sodium phosphate, pH 7.5, 100 mM KCl [13], [25] (phosphate assembly buffer). The experimental setup has been reported previously [5]. Vimentin was assembled in phosphate assembly buffer at 0.2 mg/ml for about 25 min. Then, the sample was diluted 40 to 60 fold in the same buffer and applied to the surface. We use 100 µm long cantilevers with oxide sharpened silicon nitride tips (type NP-S from Digital Instruments, Santa Barbara, USA). All AFM image were recorded in tapping-mode using Nanoscope IIIa running with software version 5.12r3 (Digital instruments, Santa Barbara, USA) [5].

### Image processing

Using the Nanoscope III software, the images were flattened and zoomed to a pixel size of about 1∶50 of the estimated persistence length of the filaments using the software supplied with the AFM (Nanoscope ver. 512r3, Veeco Santa Barbara CA). These images were opened and scaled in ImageJ V1.31v (National Institutes of Health, USA; freely downloadable from http://rsb.info.nih.gov/ij). Filament contours were traced using the “freehand line tool” and exported as XY-coordinate sets. The exported contours were smoothed using the weighted average of five contiguous XY-coordinates centered around a given XY-coordinate, thus minimizing the errors due to the discretized contours [5]. Each smoothed backbone was split into segments of increasing contour lengths in 0.1 µm increments. For each segment length *s*, the mean cosine of the angle between the initial and final directions (equation 4), the mean-square angle (equation 5), the normalized mean fourth power of the angle (equation 6) and the mean-square end-to-end distance (equation 7) were computed [8]. The normalized autocorrelation function was calculated by equation 8. Curve fitting was done in Origin 7.0 (OriginLabs Corp, MA, USA).

### Data analysis

The equations used were derived in [8], [10], and summarized in [5]. In brief:

The normalized probability distribution function in two dimensions for the filament bending angle *Θ* between segments of the polymer separated by a contour distance *s* is Gaussian and can be written as: 
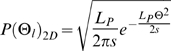
(3)


The distribution function can be used to determine the persistence length from the average cosine of the angle *Θ* between tangent vectors of the polymer:
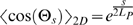
(4)


The ratio of the contour distance *s* to the persistence length equals the mean–square bending angle:
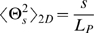
(5)


The mean-fourth power of the angle, as a statistical test of Gaussian distribution, is:
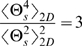
(6)


The mean-square end-to-end distance on a two-dimensional surface as a function of contour distance *s* depends on the persistence length *L_P_* according to Rivetti et al.[10]: 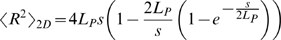
(7)


The normalized correlation function of the bending angle *Θ* along the contour is computed as:
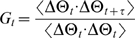
(8)


## Results

### AFM images versus Brownian dynamics simulation of IFs adsorbed to different surfaces

**Figure 1:**
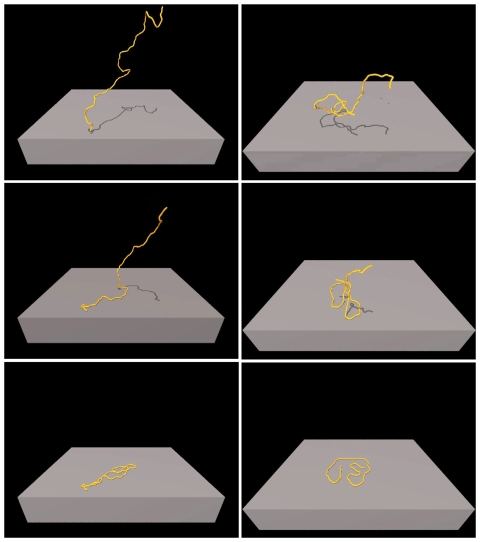
Snapshots of the two simulated adsorption scenarios. Filaments were first equilibrated in 3D. Left images illustrate the case of strong chain-surface interaction. Right images illustrate the case of weak chain-surface interaction. The upper images show the point of first contact between chain and surface (time 0 ms). The middle images show snapshots during the deposition process (time: 2 ms left image; 4 ms right image). The lower images show conformations of the fully adsorbed chains (time about 8 ms).

**Figure 2:**
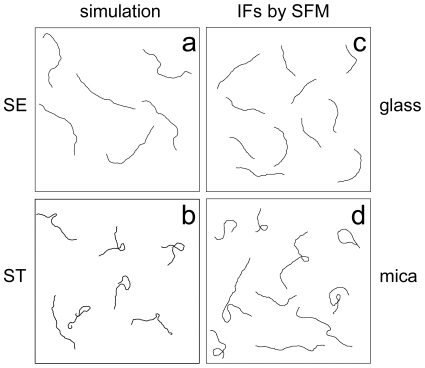
Outlines of simulated chains and traced contours of Ifs. The contour lengths of the simulated chains was set to 4 x *L_P_* and of the IFs 3 x to 7 x *L_P_*, respectively. Brownian dynamic simulations were performed for weak (a) and strong (b) filament surface interaction. If weak filament surface interaction was taken into account smooth contours with random bending can be observed (a). If strong filament surface interactions were taken into account (b) loops can be found and the curvature does not appear random. Traced vimentin IF contours deposited on modified glass (c) and freshly cleaved mica (d) are shown. After image processing (see Material and Methods), representative contours have been selected and arranged in an image. Image size: 10 x *L_P_*.

A schematic view of the deposition process simulated by the BD model is shown in Fig. 1 and 2. Two scenarios have been simulated: Fig. 1a illustrates the case of strong chain-surface interactions (ST) and Fig. 1b the case of weak interactions (SE). QuickTime movies of the two simulations are available in the supplement (Movie S1, S2).

A first qualitative comparison of the adsorbed 2D conformations after the two different deposition scenarios, using simulated and AFM-measured contours, is shown in Fig. 2. For better presentation, the scale of each plot is set to ten times the persistence length. In Fig. 2a outlines of the simulated fibers in the limit of SE are presented; Fig. 2c shows outlines of AFM images of vimentin filaments on a glass surface, where rearrangements occur after adsorption. Qualitatively, both images show similar, random conformations, suggesting that the vimentin filaments may be represented by a WLC-model. Fig. 2b shows BD-simulated contours for the case of ST. Correspondingly, outlines of AFM images of vimentin filaments on mica are shown in Fig. 2d. Again, the simulation and the experiment are qualitatively similar and clearly different from the filaments that can rearrange after deposition. Typically, loops can be found in the case of ST, and the filaments show stretches of rather uniform curvature. Sometimes long straight segments can be found followed by a narrow curve or loop. From these data one might expect that the Gaussian distribution of bending angles, which governs the WLC statistics of a homogeneous elastic polymer, is lost.

### Quantitative comparison of AFM images as BD-simulated chains under surface equilibrium and trapping conditions

**Figure 3:**
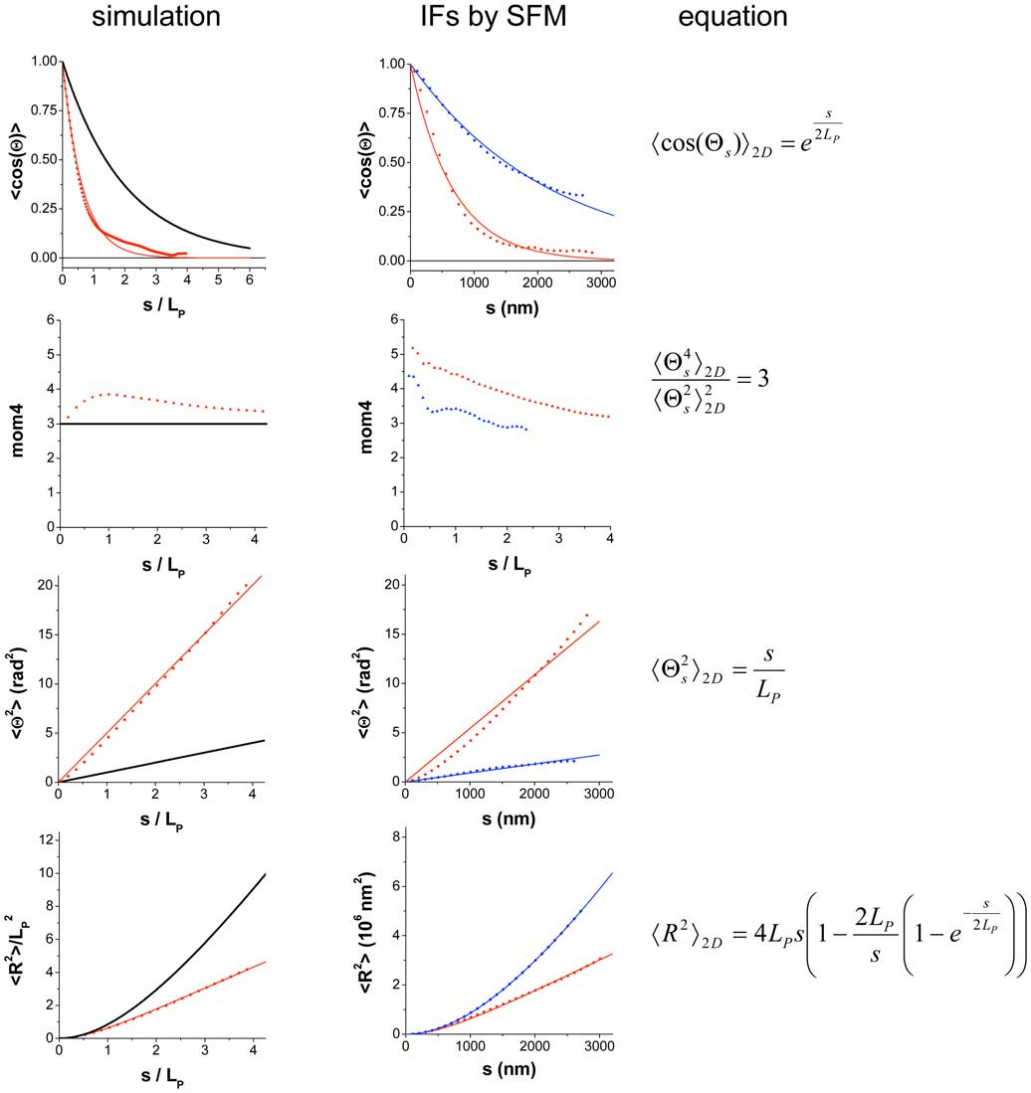
Quantitative analysis of the simulated and the traced contours from AFM images. Data points are calculated from the contours (black, simulated SE; red, simulated ST; blue, vimentin on glass; red vimentin on mica). Each data set was then fitted by the equations on the right column (straight lines). The fitting results are further described in Tab. 1.

To extract parameters that can be used to compare the fibers quantitatively, we plotted the cosine of the mean-angle, the fourth moment, the mean-square angle and the mean-square end-to-end distance against the filament contour length, and fitted these data with the equations given in the theory part. The results are shown in Table 1, 2 and Fig. 3. For visual comparison the experimental and the simulation results were merged (Figure S1). As a first general result, it is evident that all curves, including the trapped ones, can be fitted with the 2D equations. Moreover, the fourth moment of the distribution is about or more than 3, still indicating a Gaussian distribution for each deposition scenario. Only, for the mean-square angle calculation, the fit deviated systematically. Here the lowest apparent persistence length was obtained (see below).

**Table 1:** Polymer statistics of filaments adsorbed to different supports^a^.

interaction	surface	s/*L_P_* b	<s> [nm]	*L_P_*(cos(Θ)) [nm]	<Θ^4^>/<Θ^2^>^2^	*L_P_*(Θ^2^) [nm]	*L_P_*(R^2^) [nm]	counts
Weak	glass	2–4	2620	990	2.9–3.1	980	1110	173
weak	glass	3–6	3630	1090	2.8–3.4	1100	1050	56
strong	mica	0.6–1.5	1020	336	2.5–2.7	350	445	55
strong	mica	2–4	2670	310	2.7–3.5	290	370	57
strong	mica	3–6	4050	330	3.2–4.3	180	320	57

a For each support, we have computed the apparent persistence lengths from equations 4 to 8. The mean errors were less than 5% for all calculated persistence lengths.

b Range of the selected length s of the filaments divided by the persistence length *L_P_* (Assumption: full equilibrated).

**Table 2:** Polymer statistics of the simulated deposition scenarios in comparison to the normalized apparent persistence length of obtained filaments adsorbed to different supports.

surface	<s>*L_P_* a	*L_P_*<cos(Θ)>/*L_P_*	*L_P_*<Θ^2^>/*L_P_*	*L_P_*<R^2^>/*L_P_*	correlation
simulated	filaments^b^	*L_P_* = 1			
strong	1	0.44	0.37	0.44	Yes/0.02
strong	2	0.40	0.29	0.39	Yes/0.04
strong	4	0.32	0.20	0.32	Yes/0.10
Vimentin	*L_P_* = 1050 nm				
Mica	1.0	0.32	0.33	0.42	Yes/0.02
Mica	2.5	0.30	0.28	0.35	Yes/0.07
Mica	3.9	0.31	0.17	0.30	Yes/0.12

All mean errors were less than 1% in case of simulated filaments and less than 5% for the IFs. No correlation was found for the equilibrated vimentin filaments. The integral is 0.00 for both lengths.

a Length s of the filaments divided by the persistence length *L_P_* (assumption: full equilibrated); *L_P_* is the mean value of all persistence lengths measured with weak filament-protein interaction.

b 2000 counts.

### Trapping phenomena obtained by Vimentin imaging

In the case of SE, the obtained mean value for the persistence length of vimentin *L_P_* (<cos(Θ)>, <Θ^2^> and <R^2^>) is 1050 nm (Range: 980–1100 nm). In contrast to these values, the mean *L_P_* in the case of ST is 326 nm (Range: 180–445 nm) (see Tab. 1). The observed range of the *L_P_* values in the case of SE (as expected for a WLC) is independent of the filament length and the equation used. However, in the case of ST a strong dependence of the *L_P_*(Θ^2^) and *L_P_*(R^2^) of the filament lengths was measured. Hence, one may ask whether the trapping scenario observed is ideal or not, in the sense that some movement on the surface after adsorption is still possible. Dependence of the calculated persistence length on filament length was not observed with the values of *L_P_*(cos(Θ)). For long chains the lowest *L_P_*(Θ^2^) was measured, i.e. 180 nm. This is only 17% of the *L_P_* obtained in the case of SE. To understand this type of capture mechanism in more detail we performed BD simulations to mimic the ideal trapping scenario.

### The ideal trapping phenomena observed by BD simulations

**Figure 4:**
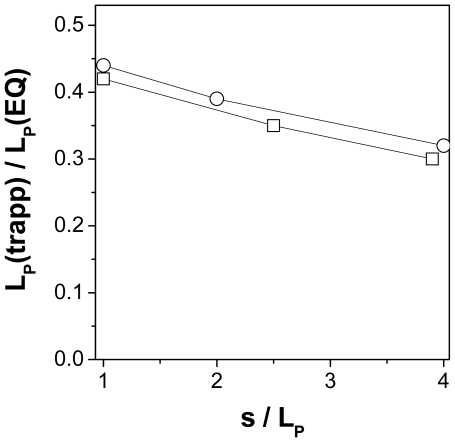
The influence of the filament lengths to the deposition processes. The normalized *L_P_* (R^2^) (values see Tab. 2, right column) are plotted versus the normalized filament lengths (squares, vimentin filaments adsorbed on mica; circles, simulated filaments (ST)).

Ideal trapping absorption phenomena (ST) were simulated on sets of 2000 filaments for three different filaments lengths. All calculated values for the apparent persistence length of adsorbed filaments with different contour lengths exhibit the same trend: the shorter the filaments, the higher the observed apparent persistence length (Tab. 2, Fig. 4). Interestingly, *L_P_*(Θ^2^) is much lower than compared to the other calculated persistence lengths. For filament lengths of *4L_P_* only 20% of the *L_P_* was calculated. This behavior is comparable to that of vimentin filaments on mica (17% of the *L_P_*).

### Normalized correlation function of the bending along the outlines

To get a robust criterion for the filament-surface interaction scenario we decided to analyze the correlation of the bending direction along the contour (Materials and Methods, eq. 7). For a kinetically trapped WLC a characteristic feature is the presence of loops due to self crossings. In fact, many loops were found in the ideal trapping scenario at contour lengths of about *4L_P_*. A loop is a conformation where the direction of the bending is correlated over a certain distance. In contrast, for an equilibrated WLC (SE) one major assumption is that the direction of the bending is not correlated. Therefore, in an ideal equilibrated scenario the theoretical value for the correlation of the bending direction is zero for all segment lengths. For the trapping scenario 2000 deposition processes were simulated with filament lengths ranging from *L_P_* to *4L_P_*.

**Figure 5:**
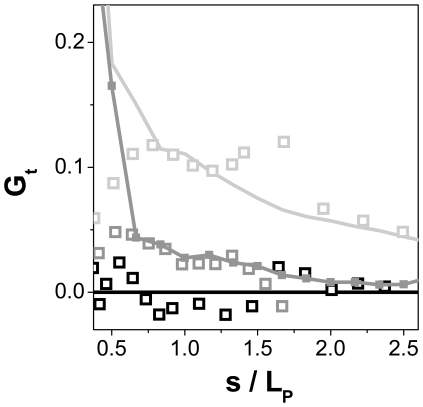
The correlation of the bending angles along the filament axis for different segment lengths. The mean length of measured trapped filaments (open squares) was 1 x *L_P_* (dark grey) and 4 x *L_P_* (light grey). Filaments with lengths of about 4 x *L_P_* adsorbed on glass (black squares) exhibited no systematic error compared to the theoretical value for equilibrated filaments of 0 (black line). The simulated filaments exhibit a correlation of the curvature by ST (line, light grey). The same correlation is obtained for vimentin filaments adsorbed on mica.

The normalized correlation function for the *4L_P_* filaments exhibits values of 0.05 to 0.12 for normalized segment lengths in the range of 0.75<(*s*/*L_P_*(Θ^2^))<2.0 (Fig. 5). For very short segment lengths of the filaments, *s*<0.75 *L_P_*(Θ^2^), the measured and the simulated curves differed significantly (Fig. 5). In that range, the deviation is due to the discretization of the contours and the subsequent smoothing. Consequently this range was excluded from the integration. The value of the integral over the range of normalized segment lengths range is 0.10 (Tab. 2) for the *4L_P_* filaments. For filament lengths in the range of *L_P_* a lower integral value of 0.02 was obtained. The kinetically trapped vimentin filaments exhibited comparable integral values. For the long filaments (3.9*L_P_*) the observed integral is 0.12 and 0.02 for the short filaments.

## Discussion

We have analyzed the conformation of vimentin filaments on surfaces by atomic force microscopy (AFM), using adsorption conditions for near-ideal surface equilibration (SE) or surface trapping (ST). SE conditions imply that the interaction with the surface is strong enough to bind the molecule to the AFM support, but weak enough to allow sliding on the surface and adaptation of a two-dimensional equilibrium conformation. ST conditions, on the other hand, prevail when the interaction with the surface is strong enough to prevent an adsorbed part of the molecule from moving further; in that case, the conformation on the surface is close to a two-dimensional projection of the three-dimensional solution conformation.

These two adsorption conditions, SE and ST, were used in Brownian dynamics simulations of the deposition of flexible polymer chains on a surface. The apparent polymer flexibility, expressed as a persistence length, was then calculated from both the experimental and the theoretical data sets using three different procedures as described above: calculation from the mean-square end-to-end distance as function of contour length, from the decay of the average cosine of the bending angle with the contour length, and from the mean-square bending angle as a function of contour length. Under SE conditions all three procedures gave the same value of about 1 µm for vimentin IFs. ST conditions consistently led to a reduced apparent persistence length, with a ratio of *L_P_*(ST)/*L_P_*(SE) decreasing from 0.45 for the shortest contour lengths (*L_P_*) to the theoretical value of 0.3 for long contour lengths (*4L_P_*). These ratios were obtained both for the data from AFM images and the simulated polymer contours (Fig. 4).

We conclude that neither the three methods used to analyze the imaged contours nor a comparison between them can distinguish between different adsorption scenarios without prior knowledge of the true value of the persistence length. However, trapping leads to a visible increase in the number of ‘looped’ conformations where the filament is bent into the same direction over long stretches. This tendency to keep the bending direction can be quantified by computing the autocorrelation function of the bending angle (Fig. 5). Trapped chains exhibit a non-zero value of this function over a large range of contour lengths, both in the simulated and in the experimental data. For surface-equilibrated chains, the theoretical value of this function is zero, which is in complete agreement with the experimental data. In addition, calculation of the apparent *L_P_*(Θ^2^) and *L_P_*(R^2^) can be used to detect the adsorption mechanism. In the case of ‘ideal SE’, *L_P_*(Θ^2^) and *L_P_*(R^2^), give similar results, whereas *L_P_*(Θ^2^) will be significant lower than *L_P_*(R^2^) in the case of trapping (since trapping results in tighter bending on the average, this difference may be partly due to the fact that eq. 5 is an approximation for the limit of small bending angles). This latter strategy allows one to determine the adsorption scenario beforehand and then to select the appropriate equation for obtaining the value of the persistence length.

## Supporting Information

Figure S1 Comparison (overlay) of the quantitative analysis of the simulated (lines) and the traced contours from AFM images (squares). Data were taken from Fig. 3. The equilibrated filaments (black) and the trapped filaments (gray) show very good agreement.
(4.25 MB DOC)

Movie S1 Brownian dynamics simulation of chain deposition with surface equilibration (see text)
(16.21 MB MPG)

Movie S2 Brownian dynamics simulation of chain deposition with surface trapping (see text)
(15.61 MB MPG)
